# Motivational context for response inhibition influences proactive involvement of attention

**DOI:** 10.1038/srep35122

**Published:** 2016-10-12

**Authors:** Zachary D. Langford, Hanne Schevernels, C. Nico Boehler

**Affiliations:** 1 Ghent University, Department of Experimental Psychology, Ghent, Belgium

## Abstract

Motoric inhibition is ingrained in human cognition and implicated in pervasive neurological diseases and disorders. The present electroencephalographic (EEG) study investigated proactive motivational adjustments in attention during response inhibition. We compared go-trial data from a stop-signal task, in which infrequently presented stop-signals required response cancellation without extrinsic incentives (“standard-stop”), to data where a monetary reward was posted on some stop-signals (“rewarded-stop”). A novel EEG analysis was used to directly model the covariation between response time and the attention-related N1 component. A positive relationship between response time and N1 amplitudes was found in the standard-stop context, but not in the rewarded-stop context. Simultaneously, average go-trial N1 amplitudes were larger in the rewarded-stop context. This suggests that down-regulation of go-signal-directed attention is dynamically adjusted in the standard-stop trials, but is overridden by a more generalized increase in attention in reward-motivated trials. Further, a diffusion process model indicated that behavior between contexts was the result of partially opposing evidence accumulation processes. Together these analyses suggest that response inhibition relies on dynamic and flexible proactive adjustments of low-level processes and that contextual changes can alter their interplay. This could prove to have ramifications for clinical disorders involving deficient response inhibition and impulsivity.

Successful motor control is dependent on the interacting dynamics of activation and inhibition mechanisms. The latter mechanisms play a fundamental role in typical and in atypical cognitive functioning, e.g. in attention-deficit hyperactivity disorder (ADHD), schizophrenia, and Parkinson’s disease^1^. The stop-signal task is a highly influential response-inhibition paradigm, which has been developed to characterize the behavioral components of motor inhibition, in particular the stop-signal response time (SSRT)^2^, and to investigate the neural processes involved^3^.

Research has focused mainly on mechanisms triggered by the stop-stimulus, dubbed reactive stopping, for which a network of brain areas has been identified as being relevant. The most influential instantiations of this network recruit the right inferior frontal gyrus, the pre-supplementary motor area, and the subthalamic nucleus^1^,^4^, and are stop stimulus activated. A parallel line of research has shifted focus towards preparatory inhibitory mechanisms, in part because of their ecological relevance^4^,^5^,^6^, and possible derailment in disorders such as ADHD^7^. The hallmark of this proactive form of inhibition is in delayed response times in situations where outright stopping *might* be required. A delayed response to a go-stimulus increases the probability of successful inhibition for any given trial^8^ and preparatory processes are generally believed to benefit reactive inhibition^9^. Moreover, it has been shown that response speed can be adjusted on a very short time scale^5^ and that it is possibly related to the computation of a trial-wise (subjective) expectation of encountering a stop-signal in the upcoming trial^10^. Experimental studies have suggested that proactive response inhibition recruits the same reactive response-inhibition network as described above, which then implements partial instead of complete inhibition^4^,^11^,^12^. Given the wealth of converging evidence, this is one likely explanatory mechanism in the variation seen in proactive inhibition, but not necessarily the only explanation, or the sole mechanism involved^11^,^13^,^14^,^15^.

It is typically assumed that in both reactive and proactive inhibition behavior ultimately depends on the action of a central response-inhibition module^16^. Yet, computational work suggests that a large portion of the time needed to implement response inhibition is taken up by non-inhibitory processes related to the processing of the stop-stimulus^17^,^18^,^19^, and some recent experimental work has varied inhibition demands while controlling for such processes^20^,^21^ (suggesting a less modular system^22^). Parallel to reactive control^23^,^24^, in the domain of proactive inhibition the involvement of attentional processes has recently been emphasized^11^,^19^,^25^,^26^. In a first relevant study, magnetoencephalographic data showed that the attentional processing even at the level of the go-stimulus of a stop-trial varies in a way that affects behavior, in that it is enhanced when response inhibition is ultimately unsuccessful^27^. In this same vein, a recent electroencephalographic (EEG) study of ours showed evidence that for go-trials the inferoposterior N1 component (an index of selective attentional processing^28^) was being systematically down-regulated as response times were slowed, but only when outright stopping was contextually relevant^29^. Since this work focused on go-trials, it clearly relates to proactive response inhibition, meaning that the respective fluctuations in RT and attention are cautionary and preparatory in nature, rather than being related to outright inhibition.

In addition to the involvement of attention in response inhibition, there is a growing body of evidence implicating early attentional processes in reward-related processes^30^,^31^,^32^,^33^, as well as in their interaction^34^. Consistent with this convergence on early attentional processes, it has recently been shown that reward can also play a modulatory role, in particular in measures of reactive response inhibition^35^,^36^. We have recently reported a series of studies in which associating reward with one of two possible stop-stimulus colors also led to shorter SSRTs, despite the fact that any difference in global preparation was precluded since reward-related trials were presented in a random sequence together with all other trial types^37^,^38^. In an EEG version of this experiment, we have demonstrated that enhanced attention to the reward-related color seems to play a role in bringing about the reward-related SSRT benefit^39^. Furthermore, this experiment featured task blocks that were devoid of any reward associations. Using these trials as a comparison, we demonstrated that the sensory/attentional N1 component to the go-stimuli of stop-trials was enhanced throughout the reward-related block. Given that reward was exclusively related to stop-stimuli, we considered this effect on the go-stimuli (of stop-trials) a context effect in the sense that attention is increased globally^40^. Currently we investigated contextual effects of rewarding successful reactive response inhibition on proactive inhibition; although this is a indirect as far as motivational effects on proactive inhibition goes, it circumvents the problem that simply rewarding a cautious response mode will lead to rather trivial response slowing.

The present study used single-trial EEG analyses to model the covariation of the visual N1 with response time framed within the context of proactive response inhibition. We focused on go-trials and the relationship between the sensory/attentional N1 component and response time in rewarded-stop (RS) and standard-stop (SS) task blocks (see Fig. 1c). For the SS blocks, we anticipated to replicate our earlier results of a systematic relationship between single-trial response times and N1 amplitude^29^. For the RS context, however, we hypothesized two different possible patterns of results: **(1)** The relationship between response times and N1 amplitudes could be similar or even more pronounced, given that we consider it a marker of proactive response inhibition, which is generally a useful process when trying to successfully inhibit a response^9^, and could hence be more strongly engaged in a task context in which successful inhibition can be rewarded. **(2)** The global attentional increases driven by reward context^39^,^40^ might interfere with or abolish the relationship between visual attention and response speed. This might be particularly relevant here because of the rapid temporal succession of go- and stop-stimuli, which might preclude a fully specific reward effect on stop-stimuli without simultaneously also enhancing the processing of go-stimuli presented at the same spatial location. We further modeled the behavioral data as an evidence accumulation process to pick apart the perceptual decision making dimensions that are relevant for differentiating between the SS and RS task blocks (see refs 5, 11 and 41 for further motivation related to response inhibition, and see ref. 42 specifically for the relationship between RT and stopping behavior in a drift-diffusion framework). Specifically, we were interested to see whether evidence accumulation would proceed faster in a reward context, which would be consistent with an attentional increase to go-stimuli.

## Results

### Single-trial EEG analyses

#### Attentional covariation with response time

**Figure 1:**
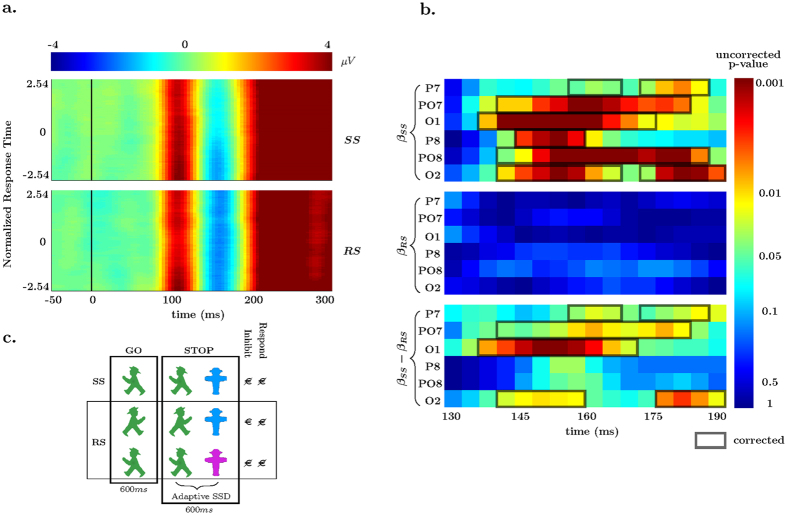
Response Time Effects. (**a)** Averaged N1 EEG electrodes (O1, O2, PO7, PO8, P7, P8) data plotted by normalized (per individual) response times in the standard-stop context (top) and rewarded-stop (bottom). (**b**) Uncorrected p-values for the standard-stop (SS, top) 1-sample t-test, reward-stop (RS, middle) 1-sample t-test, and paired-sample t-test (bottom) for six electrodes. Significant temporally corrected values at *α* = 0.05 are overlaid. (**c**) Stimuli and paradigm (flanking stimuli are not shown) showing go-trials, stop-trials, and potential payouts for stop-trials in both the SS and RS blocks. The color coupled to reward (blue in the example) for a successful inhibition was instructed before the block began.

As a first exploration the average of all N1-related electrodes were plotted as a function of individually normalized response time in Fig. 1a. These data were time-locked to the onset of the go-signal of *correct* go-trials. A clear N1 effect is seen within the analysis window (130 to 190 ms). This N1 effect visibly dampens as RT increases in the SS context, but is sustained across RT in the RS context. To explore this quantitatively a robust multilevel single-trial EEG methodology was applied in two separate steps. This is an optimal method as it can account for both categorical (context) effects and parametric (RT) effects within a unitary model. In the first-level statistical models regression parameters were estimated for each individual at each of the inferoposterior N1 electrodes for each time-sample between the defined time window. These included linear predictors for the categorical RS and SS factor, continuous response time per context, and noise, for each of the 6 electrodes and for each sample point.

#### Standard-stop go-trials and response time

Covariational RT and EEG differences within the SS context were tested using a bootstrapping 1-sample t-test procedure to ‘synthesize’ individuals’ *β* parameters for statistical testing at the second level of analysis. This is a test that probes the relationship between RT and N1 amplitudes. Within the N1 electrodes chosen for analysis, a clear pattern emerged in the SS trials. As RT slowed in the SS context the inferoposterior N1 voltages systematically became less negative across all individuals. Note that while the analysis is performed separately for multiple time-points, correcting for multiple comparisons using a 1-dimensional temporal cluster correction showed evidence that such an effect was indeed present in all analyzed electrodes and peaked around 160 ms (see *β*_*SS*_ of Fig. 1b).

**Figure 2:**
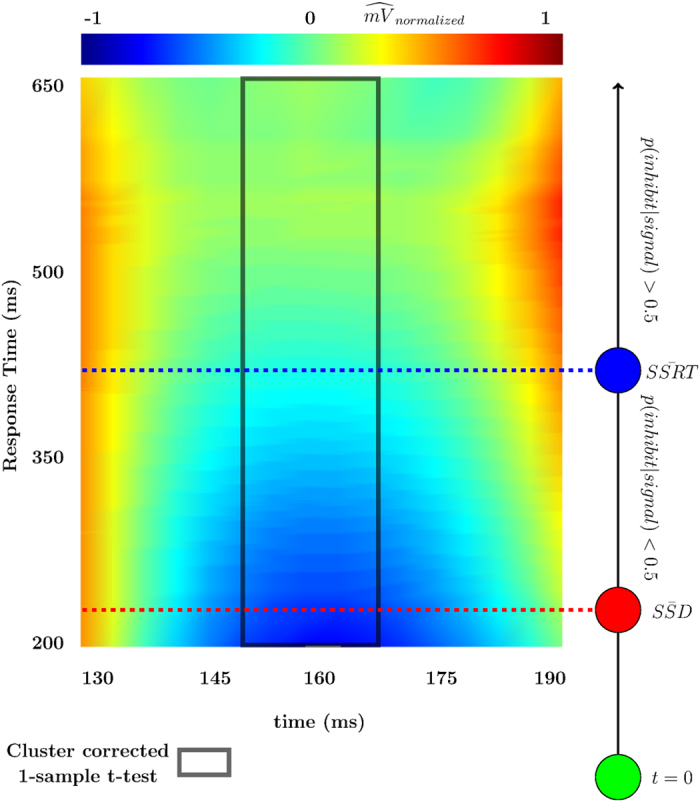
Model Prediction. Predicted voltages individually normalized and then averaged across individuals plotted by response time and time since go presentation. Electrode plotted for each individual was the electrode from the optimized *r*^2^ vector. Significant cluster from 1-sample t-test overlaid on top. To the right the relationship between SSRT, SSD, and *p*(*inhibit*|*signal*) are shown in relation to go-RT and the single trial N1.

Further testing was done by constructing an optimized electrode vector from each individual’s electrodes using the maximal *r*^2^ as the decision criteria for inclusion (see ref. 43 for the motivation behind optimized averaging in EEG research). The same 1-sample t-test was run on this optimized vector of electrodes to support the previous finding. Cluster-corrected significant differences were found from ~148 ms until ~167 ms. The model was then inverted and the predicted voltages given by this optimized electrode vector were normalized for each individual and subsequently collapsed across individuals to develop a predicted-voltage plot of RT * post-go time * voltage to visualize the effect (see Fig. 2). Included in Fig. 2 is an overlay that depicts the relationship between RT and the probabilities of either successful or unsuccessful inhibition (as predicted using the horse race model^2^).

#### Rewarded-stop go-trials and response time

Go trials from the RS blocks were submitted to the same second level covariational approach used to test the *β* parameters in the SS blocks. The N1 attenuation effect as a function of RT seen in the SS task was completely absent from the RS blocks. The p-values for the 1-sample t-test are shown in the middle plot (*β*_*SS*_) of Fig. 1b. This indicated that there was a lack of evidence for a covariational relationship between RT and the N1 at a single trial level (i.e., accept null *β* = 0, even in the absence of multiple comparison corrections).

#### RT differences between contexts

To test that the relationship between RT and N1 amplitude seen in the 1-sample t-tests are more than qualitatively different between SS and RS contexts, a paired-samples bootstrapping t-test was applied to the RT beta coefficients. Four of the six electrodes were shown to be different between the RS and SS contexts using the same 1-dimensional temporal cluster correction method, as shown in the bottom panel (*β*_*SS*_ − *β*_*RS*_) of Fig. 1b. To summarize the covariational RT effects we observed a relationship between single-trial N1 amplitude and response speed, with longer response times being related to slower behavioral responses, similar to what we have observed in an earlier study in a standard stopping context^29^. Importantly, this relationship was exclusive to the SS and absent in the RS context.

#### Categorical differences between contexts

**Figure 3:**
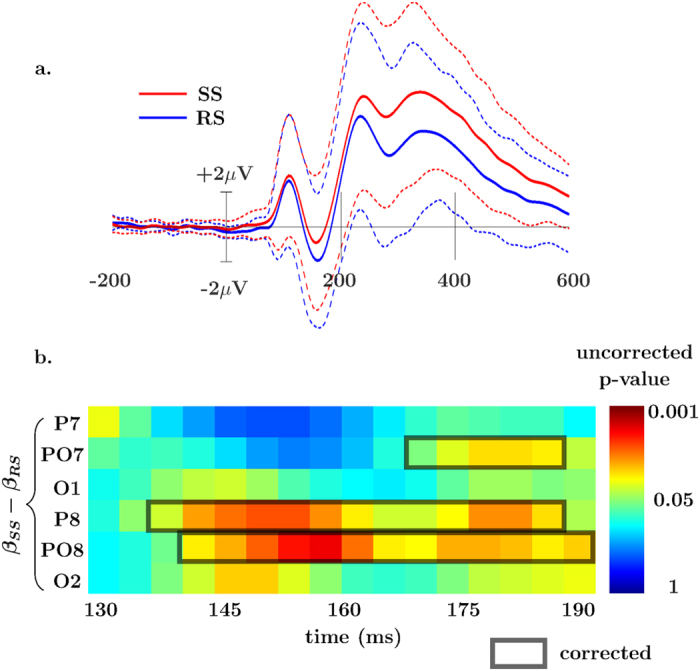
Categorical Effects. (**a)** Standard ERP of all 6 N1 electrodes averaged with 95% confidence interval. (**b)** Time course of p-values from the paired samples t-tests with the tested range of 130 to 190 ms post-go presentation for the categorical *β* parameters of the SS and RS blocks.

Group-level differences in the categorical factors RS and SS were tested using the same paired samples bootstrapping t-test. This is generally related to a standard ERP, but controlling for RT, and additionally modeling noise. For reference to a common (ERP) analytic scheme, the standard ERP is shown averaged over the 6 N1 electrodes in Fig. 3a. For the paired samples t-test all 6 of the electrodes tested showed more negative amplitudes in the RS N1 at an uncorrected threshold of *α* = 0.05, as is seen in Fig. 3b. After controlling for multiple comparisons using a 1-dimensional cluster correction, 3 of the 6 were found to be different (specifically PO7, P8, and PO8). These effects started at ~140 ms post-stimulus and continued (for some electrodes) until the end of the tested interval of 190 ms. Go-trials in the RS blocks had more negative amplitude than in the SS context. This result extends our earlier observation in this dataset of an enhanced N1 ERP amplitude for the go-stimuli presented during stop-stimuli^39^.

#### N2-P3 complex

**Figure 4:**
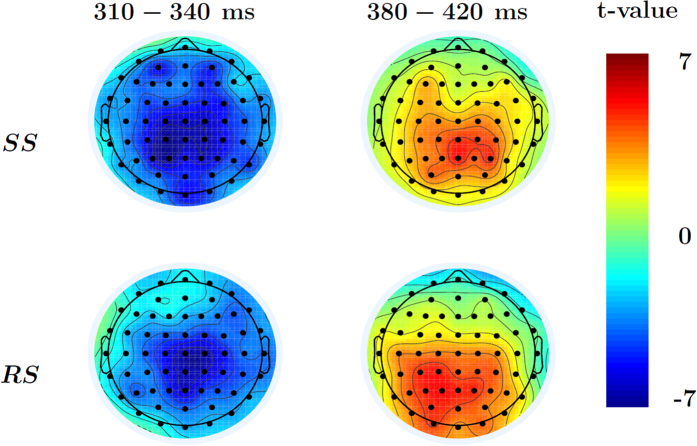
1-sample t-test on EEG-response-time relationship between 200 and 600 ms. t-values from 1-sample RT *β* t-tests averaged within the significant cluster corrected time ranges for both the early and late components. The data indicate a negative relationship between RT and EEG data in the N2 range, and a positive one in the P3 range, albeit only for the RS context after multiple-comparison correction.

While the N1 component was our explicit a-priori focus of interest, we temporally extended our analysis of possible EEG-response-time covariation in the same fashion to identify the possible involvement of later neurocognitive mechanisms. The same model was applied to the full scalp in the window of 200–600 ms after the go-signal in correct go-trials separately for the SS and RS task context. Cluster-corrected 1-sample t-tests of the RT parameters showed evidence of a covariational effect peaking around ~320 ms in both the SS and RS blocks, and a later effect peaking around ~400 ms in the RS blocks. The earlier component in both blocks was a negative wave (N2) and had a fronto-central scalp distribution (Fig. 4b), and became more negative with increased RT. The later RS component was a positive-going wave (P3) with a central-posterior scalp distribution (Fig. 4b), and became more positive with increasing RT. A paired-samples t-test was done on both the categorical and RT *β* parameters to test for differences between the SS and RS contexts. No differences were found after cluster correction for either paired-samples test. Therefore, as opposed to the N1 component, neither the go-locked N2 nor P3 were sensitive to reward availability for stopping, while also showing some relationship to RT that was largely independent of the two different task contexts.

### Behavioral Analysis

#### Standard analysis

Correct go-trial mean RTs were similar in the RS block (426.5 ± 9.5 ms) compared to the SS block (420.3 ± 8.6 ms). Go-trial accuracy in the RS block (96.6 ± 1.1%) was also similar to the SS block (9.96 ± 0.6%). The average stop-signal delay in the SS blocks was 229.3 ± 9.4 ms, and the average SSRT in the SS blocks was 177.3 ± 4.2 ms (reproduced here for Fig. 2, see ref. 39 for further details related to SSRT, stop-trial behavior, and other calculations not pertinent to the current analyses).

### Hierarchical Drift Diffusion Model

Drift-diffusion models incorporate a framework for forced choice decisions that can be used to account for accuracy and response times in a given trial. Models were fit using a hierarchical Bayesian estimation scheme to sample from parametric distributions corresponding to the rate of sensory/perceptual accumulation of go-stimulus information (drift, *v*), level of response caution (response threshold, *a*), and the combined time needed for nondecision processes (nondecision time, *t*_0_). Model selection for the HDDM started by fitting a null model, i.e. ignoring differences between go-trials from the SS and RS contexts. SS and RS differences in response threshold, drift, and nondecision time were then successively added to the model, and in combination (by taking away the initial constraints of equality across SS and RS). Table 1 reports the improvement in model fit of Deviance Information Criterion (DIC) for the three best hypothetical models considered, as well as the null model. Based upon DIC, the model with all parameters included was selected for further analyses.

As seen in the bottom of Table 1, the differences between the parameter distributions in the RS and SS are different (as seen in the high probabilities that the sampled distributions are dissimilar) for the model that includes all parameters. The RS blocks, relative to the SS blocks, have a raised response threshold, a steeper drift, and also a smaller amount of time devoted to nondecision processes (*t*_0_). Notably, the standard mean and accuracy analyses above had indicated very similar go-trial behavior in the two task blocks. The DDM analysis indicates that this is brought about in different ways, in which in RS blocks short nondecision times and faster drift-rates are compensated for by a raised response threshold in order to maintain a similar response speed, as participants were instructed to do.

## Discussion

The current EEG study investigated neural processes underlying proactive response inhibition during the stop-signal task in human subjects and focused on early attentional mechanisms. The analysis was based on a comparison of go-trials from different trial blocks in which successful response inhibition was either explicitly motivated by reward prospect, or not, thereby probing for differences due to motivational context. We found a significant relationship between the single-trial amplitudes in the attention-related visual N1 component and response speed in the standard stop-signal task, but not in the rewarded-stop task blocks. This was accompanied by an overall more pronounced N1 in the reward context. Additionally, we found evidence for relationships between RT and EEG in the N2 and P3 time-ranges, which however did not differentiate clearly between the two motivational task contexts. Finally, despite overall mean behavior being highly similar across blocks, we observed differential results of a drift diffusion analysis, in which the mere difference in context led to differences in all key parameters. Specifically, a stop-trial-related reward context modulated go-trials to have higher drift rates and lower nondecision times, together with a elevated decision threshold.

The classic inferoposterior N1 component is thought to index the selective attentional processing and discrimination of the visual stimulus in mid- and high-level visual areas, and to generally indicate how much attention is paid to a visual stimulus^28^,^44^. The single-trial ERP analysis demonstrated that as response times increased, the amplitudes of this component decreased, albeit only in the SS block. The damping of N1 amplitudes as responses slow is likely an indication of a down-regulated discrimination of the go-stimulus. Mathematical modeling of the stop-signal task predicts that the slower the response the more probable successful stopping is to occur^45^. Therefore, a down-modulated go-stimulus processing, as measured by the N1 component, is arguably advantageous for later inhibition. Consistent with this notion, an analysis of stop-trials in this same dataset had indicated that the N1 to go-stimuli was larger for trials that ultimately were not successfully inhibited^39^.

These results in the SS block of the present study replicates our recent report, in which we found the same relationship between N1 amplitudes and response times for go-trials in a standard stop-signal task^29^. Additionally, this earlier work indicated that this relationship is likely under proactive top-down control because it was absent in control blocks in which stop-stimuli were task-irrelevant, which abolishes any need not just for outright inhibition but also for strategic response slowing. Notably, in this previous report this absent relationship coincided with a slightly attenuated average N1 ERP component for this stop-irrelevant task context. The present findings are important not least as a replication because our previous report was the first description of this relationship between single-trial N1 amplitudes and go-trial response times. Additionally, some factors were different in the present work, which therefore do not seem to be critical for this relationship to arise. In particular, in the present work, the target stimulus was always flanked by four distractor items, thereby increasing the need for selective attention, whereas our earlier work^29^ did not feature such distractors. Simultaneously, the present comparison between task blocks is arguably more specific than in our earlier work, in that the two blocks were very similar in many regards, and still yielded different results. Most notably, go-trial performance was extremely similar across the two blocks, so that the present differential results arise in an absence of any clear overt behavioral difference. Additionally, the two blocks did not differ strongly in task requirements. Specifically, our earlier comparison between stop-relevant and stop-irrelevant blocks compared tasks that differed quite fundamentally in requirements, in that a regular stop-signal task sets up a dual-task situation (with opposing requirements to go or stop) that the stop-irrelevant blocks did not have^5^. Based on drift-diffusion modeling we argued that it was not the dual-task nature that drove behavior in the stop-relevant blocks, in particular not on a single-trial level. The present data corroborates that the presence of the relationship between N1 amplitudes and response time is not majorly dependent on such task requirements, because those were highly comparable across blocks in the present study.

Another important aspect relates to the relationship between the categorical/ERP differences across conditions (SS vs. RS) and the within-condition variation. A very recent related report investigated the role of visual attention in proactive (inhibitory) control^25^. These authors found that attention to the go-stimulus is enhanced if this stimulus might change into a stop-stimulus (or an additional control condition requiring a double response). This was interpreted as reflecting the involvement of attentional processes in proactive control, albeit not necessarily in an inhibition-specific fashion. In addition, those authors found that attentional modulations of go-stimulus processing disappeared when stop-stimuli were auditory rather than visual. These results could be interpreted as representing an effect of monitoring a stimulus for a relevant change, much like in our paradigm. We see our categorical reward-context modulation of the N1 as a related process, in which attentional monitoring is generally ramped up in order to optimally detect a reward-relevant stimulus. In our mind, such categorical effects are highly relevant, but suggest that the within-condition variation is the aspect that is more closely linked to behavior.

Extending the analysis window beyond the planned N1 time- and electrode-range, we found evidence for a relationship between RT and EEG activity in the N2 and P3 time-range, both of which were enhanced (N2 more negative, P3 more positive) as RT increased. While the P3 effect only survived multiple-comparison correction for the RS context, there was no evidence for a real difference between the contexts for either time-range. The results therefore seem to suggest that also later processes scale with RT, without clearly differentiating between the two motivational contexts. A specific interpretation of these relationships seems difficult, given that they concern simply go-trials. Yet, given the context of the stop-signal task, one could speculate about a link to components that are typically found in response inhibition, where N2 and P3 components play a prominent role both in the Go/Nogo task and the stop-signal task^46^. Having a related signature in slow-vs-fast go-trials might speak towards additional neurocognitive processes that deliberately slow down responses. Yet, this interpretation is naturally speculative at this point.

The main contribution of the present study is delineating the effect of a reward context (for successful stop-trials) on the above relationship between attention and response time in go-trials. This contextual modulation had two clear effects. **(1)** The average N1 amplitude was enhanced for go-trials from the reward-relevant task context, probably indicating a generally increased level of attention. **(2)** There was no covarying relationship between the N1 and response time on the single-trial level. This pattern is consistent with the second hypothesis raised in the introduction, namely that a context effect of RS stop-trials generally increased the amount of attention paid also to the go-stimuli, and that this process simultaneously overrides any fine-grained relationship between attention and response speed.

Concerning the behavioral data, one thing to note in particular is that the mean response times were nearly identical in the two task contexts. Overall go-trial response time is typically a bit artificial in the stop-signal task since it strongly relies on the instruction given to the participants (and their compliance to it), i.e. if successful stopping would be the only priority, simply refraining from a button press would be the most successful strategy (typically discouraged by instruction). Along similar lines we instructed participants not to slow down their responses during RS blocks compared to the SS blocks. Yet, despite this high degree of similarity in behavior, the reward context had an effect on go-trial ERPs as well as on the single-trial relationship between RT and the N1 amplitude. This is interesting not least because reward effects often take highly specific forms of benefitting precisely and exclusively the rewarded task aspect, and since stopping performance in this task was specifically enhanced for reward-related stop-trials and not for randomly intermixed no-reward stop-trials that differ only in the color of the stop-stimulus^39^. As indicated above, these effects likely represent context effects in the sense that a reward-anticipation-related increase in attention to possible stop-stimuli automatically entails enhanced attention to the go-stimuli that always rapidly precede them (in stop-trials) or are presented in isolation (in go-trials)^25^. While this seems plausible, given the tight temporal succession of events in stop-trials, it should be noted that we and others have also speculated in the past that there is a sharing of attentional resources between go- and stop-stimuli in a given stop-trial^27^,^47^. Moreover, it is often found that attention away from a stimulus that might interfere with obtaining a reward is generally considered a feasible mechanism e.g. in delayed gratification context^48^. It seems possible that a reward context also changes these relationships.

As highlighted in the previous section, standard go-trial performance was highly similar across the two task contexts. Yet, looking at drift diffusion modeling, we found that there were in fact subtle changes in the sub-processes of evidence accumulation that jointly determine behavioral outcome. Specifically, both drift rate increased and decision threshold increased, while nondecision time decreased in the RS blocks. A decrease in nondecision time and increase in drift rate leads to quicker response processes, whereas the raised decision threshold led to slower, more conservative responding. Therefore, it seems that the decision threshold compensates for the changes in the other two parameters. While the reason for this might be a bit artificial, in that it likely relates to the instruction not to slow down responses across the two task contexts, it still illustrates nicely a fine-grained process structure, in which even near-identical mean behavior can arise from different constellations of different drift diffusion parameters (i.e. modeling RT distributions and accuracy is more powerful than summary measures). This finding is generally reminiscent of results from a stop-signal task that used stimuli with different image quality, where it was found that reduced drift rate and increased nondecision time for low-quality images was compensated for by an increased decision threshold^11^. It seems likely that our presented results are strongly related, just the source of variation in stimulus processing is internal rather than external, and likely strategically employed. When speculating about the relationship of these parameters to our electrophysiological results, it seems reasonable to link the N1 modulations to differences in drift rate and possibly nondecision time, in that early attentional processes plausibly map onto both the rate of and starting time of evidence accumulation, which might furthermore relate to anticipatory attentional processes that precede the actual trial (e.g., ref. 49). The increased decision threshold, in turn, might relate to a process more linked to the motor output level, which we did not capture in our analysis of the EEG data (see refs 25, 50 and 51 for a broad discussion of candidate neural signatures). Given the fact that for obtaining reward going slow would be beneficial (but was explicitly discouraged), the changes in drift rate and nondecision time might also reflect a non-instrumental context effect, which is then counteracted by an increased decision threshold.

The current manuscript provides new evidence of a strategic and dynamic modulation in the attentional processing of go-stimuli in a standard stop-signal task using single-trial ERP analysis. Go-trial amplitudes were dampened the more delayed a response. This confirms our previous work using the same general methodology^29^, and is in line with work using both a different analysis strategy (comparing go-stimulus N1 amplitudes for stop-trials in which inhibition was ultimately successful vs. not) and neuroimaging modality (MEG)^27^. Yet, under a motivational context in the form of reward for successful response inhibition, this modulation was not evident, though attention was in fact generally increased by the mere presence of the probability of a reward. This went along with subtle changes in the relationship between different drift diffusion parameters that overall still resulted in near-identical mean behavior. Together, it appears that a reward context, even if not directly relevant for the processes studied, can introduce changes in a global attentional state, perhaps towards a sustained strategic proactive control mode^40^,^52^, thereby having an impact on all stimuli and trials that are comprised in this environment. More generally, it furthermore explicitly links differences in proactive slowing to (contextual) motivational factors, hence suggesting additional possible pathological mechanisms for patient populations with deficits in proactive response inhibition like ADHD^7^.

## Methods

The analysis in the current manuscript is based on previously reported data^39^. In the present manuscript, we focus on procedures and methods pertinent only to the current analyses, which largely focus on go-trials from a task context in which successful inhibition in stop-trials would yield a reward vs. one where this was not the case, which were not analyzed for the previous report.

### Participants

Twenty healthy right-handed students participated in the experiment (6 males, 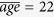). All participants had normal or corrected-to-normal vision and no history of psychiatric or neurological disorders. Written informed consent was obtained from all participants. The study was conducted in accordance with the Declaration of Helsinki and was approved by the Ethical Committee of the Faculty of Psychology and Educational Sciences at Ghent University. Participants received a base compensation of 20 euros and an additional performance-dependent bonus described in the next section. Because of a considerable number of missed and incorrect go-trials (16.5%, with a range of 1% and 9% for the remaining participants), the data of one participant were excluded from all analyses as already done in the earlier report using this dataset^39^.

### Stimuli and Procedure

Throughout the experiment a black rectangular box and a white fixation dot were presented on a grey background at the center of the screen. Go stimuli were green traffic light symbols pointing to the left or to the right. The target go stimulus was presented centrally above the fixation dot, and was additionally surrounded by two green traffic symbols on both sides that were balanced in congruency (i.e., directly next to the target was one stimulus with the same orientation and one with the opposite, again flanked by their respective mirror image) and had to be ignored. This exclusively served to globally increase the attentional load of the task, without varying the congruency level. Participants were asked to respond rapidly with the index finger (left mouse button) or middle finger (right mouse button) of their right hand according to the orientation of the central go traffic sign. A don’t-walk traffic sign was used as a stop stimulus. The color of this signal could either be blue or pink with equal proportions. In both go and stop trials the total stimulus presentation duration was 600 ms, followed by a randomly-distributed inter-stimulus interval of 1000 to 1400 ms. Participants completed two blocks, a reward block and a no-reward block, and block order was counterbalanced across participants.

Participants started with a short practice run, including 34 go trials and 20 stop trials with 10 blue stop signals and 10 pink stop signals. In stop trials the interval between a go and stop stimulus (go-stop delay) was constantly adapted to create and maintain a 50 percent rate of correct stopping. A staircase procedure was implemented that increased the go-stop delay by 34 ms after a successful stop trial (SST) and decreased it by 34 ms after an unsuccessful stop trial (UST), with a minimum of 34 ms and a maximum of 567 ms delay (starting value: 200 ms). Pink and blue stop trials shared the same staircase, which hence controlled the stopping-success rate over all stop trials within a block. Reward was only assigned to one of the stop signal colors at the start of the reward block, i.e. immediately after training for half of the participants (for whom the reward contingency was then explicitly removed at the end of the block) and after the no-reward block for the other half. Block order and the color of reward-predictive stop signals (pink or blue) were counterbalanced across subjects. Two long blocks were used to minimize possible carry-over effects related to reward-related colors^31^,^53^.

Both experimental blocks consisted of 5 runs of 100 trials each, yielding a total of 320 go trials and 180 stop trials (90 trials for each color) per block. In the reward block participants could win points for successful response inhibition in reward-related stop trials, but not in reward-unrelated stop trials. At the end of every run the amount of points gathered in that run was shown. Participants were also informed that these points would be added up at the end of the block, yielding an extra bonus of between 0 and 6 euro based on a specified transformation from points to money. Subjects were asked to respond as fast as possible and not to slow down their responses during the experiment, which is important when evaluating stopping performance^54^. Additionally, to further prevent such slowing, participants were told that the collected points in a run would be set to zero in case they significantly slowed down their responses. Since this procedure turned out to be quite effective, this correction was never actually used.

### Recording and Analysis

#### EEG recording

EEG data was collected with a 64 channel Biosemi ActiveTwo system (Biosemi, Amsterdam, Netherlands) using a standard 10–20 system, sampling data at 256 Hz. External electrodes were attached to the left and right mastoid and at the outer canti of both eyes and directly above and below the left eye. Data were re-referenced offline to the average of the left and right mastoid and a low-pass FIR filter was applied at 30 Hz (−6 dB attenuation at 33.7 Hz). Blinks were removed using independent component analysis. Go-locked epochs were made with a time window from −200 to 1000 ms, using the pre-stimulus period for baseline correction. Automatic artifact rejection was performed on these epochs with a subsequent visual inspection to reject missed artifacts. Automatic rejection removed trials with values that fell outside of −/+150 mV. Furthermore, epochs including horizontal eye movements were detected by a step function in the bipolar eye channel (with a threshold of 60 mV, window size of 400 ms and window step of 10 ms). After all rejection techniques were applied an average of 94.6 percent of epochs remained.

#### Single-trial EEG analysis of the inferoposterior N1 and exploratory extension to later time-ranges

The inferoposterior N1 locations were chosen to be closely aligned with previous work^44^. These included 6 posterior electrodes, 3 on the right and 3 on the left of a standard 10/20 EEG system (specifically, O1, O2, PO7, PO8, P7, P8). The 6 electrodes were used in further analyses to examine the visual N1 with the time range defined between 130 ms and 190 ms after the onset of a go-signal. Such inferences in principle are reverse inferences (inferring that attention was affected by looking at a neural marker without explicit modulation), yet there is a highly specific link between the N1 component and attention.

The analysis of interest investigated the relationship between single-trial EEG data and response speed on go-trials in the SS and RS blocks. To this end, single-trial EEG analyses were carried out using the package LIMO EEG^55^. At the first-level single-trial analysis fits a general linear model of the form *y*_*e,s*_ = *Xβ*_*e,s*_ + *noise* to trials of EEG data (y), separately for all analyzed electrodes (e) and sampling points (s) in the N1 time window (130 ms to 190 ms post go-stimulus). The five predictors in the design matrix X were the categorical SS and RS go-trial types, the single-trial normalized (per subject, per condition) reaction times, and a noise variable.

Two types of tests were run on the group level data as implemented in LIMO EEG: **(1)** We used robust one sample t-test that tests if the average effect (mean of *β* values) significantly differs from zero. This determines the significance and direction of *β* parameters per sample point in each context, individually. In this approach, subject beta weights are drawn randomly with replacement to provide an approximation of the t-distribution under the null hypothesis (i.e. bootstraps on centered data). **(2)** In a similar manner we computed paired samples t-tests to generate a null distribution of t-values to test *β* differences between contexts. To account for multiple comparisons, we used temporal clustering by which only clusters with a mass (sum of t values) bigger than the 95th percentile of the null distribution are considered significant. In this case, the null distribution corresponds to the maximum cluster value across electrodes measured at each bootstrap computed on the nullified data^56^.

Following these analyses, we determined the electrode for each subject individually that maximized model fit based on *R*^2^. This vector of maximized *R*^2^ electrodes was then used to run another 1-sample t-test on the *β* parameter of the RT predictor in the SS condition. To visualize this effect we plotted the model predictions by normalizing the max *R*^2^ electrodes for each individual, aligning by response time, and averaging over all individuals.

While we had strong hypotheses about the visual N1 based on past research, a more exploratory analysis was undertaken to probe other later effects known to be attention related. In this analysis we used the same procedure as with the N1 but with a broader time window (200–600 ms post-go presentation) and the full electrode montage. The same 1-sample t-tests and paired samples t-tests from the N1 analysis were used to determine differences in the population.

#### Behavioral analysis

Behavioral data was analyzed concerning standard response-time and accuracy parameters before^39^, which we reproduce here when needed. Additionally, we ran drift diffusion models. Drift diffusion models encapsulate a mathematical description of a binary choice process and are defined by three central parameters^57^, that have also have been used to quantify the decision processes of proactive inhibition^5^,^41^,^58^. These parameters are the response threshold (a), the rate of approach to the threshold, known as drift rate (v), and processes that precede and succeed the actual decision process giving rise to nondecision time (*t*_0_).

Bayesian estimation was used to model the parameters using the Hierarchical Drift Diffusion Model (HDDM) software^59^. Seven candidate models were fit to the data; a null model, a full model (including a, v, and *t*_0_), and 5 reduced models; a model without *t*_0_, a model without a, a model with only a, a model with only v, and a model with only t0. 40,000 posterior samples were taken for each model, with a burn-in of 10,000 samples, and a thinning factor of 3. Five percent of the behavioral data was assumed to come from a uniform distribution that is not adequately explained by drift diffusion processes (e.g., physiological interruption, task unrelated distractions^60^). Each model was checked for convergence using the Gelman-Rubin diagnostic^61^.

Model fit was assessed using the deviance information criterion, DIC^62^. DIC penalizes how well the model fits the data, as well as the number of parameters used to explain the data. Furthermore, posterior-predictive checks were made on each model to assess the performance and reasonableness of the model estimates and to check the models ability to reproduce the observed data. After model selection, posterior distributions were probed to determine differences directly in the parameters between the SS and RS task contexts. This is accomplished by examining the proportion of posterior samples falling above or below a two specified posterior distributions, resulting in a probability that one posterior distribution is greater or less than the other^63^.

## Additional Information

**How to cite this article**: Langford, Z.D. *et al.* Motivational context for response inhibition influences proactive involvement of attention. *Sci. Rep.* **6**, 35122; doi: 10.1038/srep35122 (2016).

## Figures and Tables

**Table 1:** Top Differences in DIC of tested model from the null model (lower DIC is better).

	model	*DIC_diffrence_*	
	Null	—	
	a, v	−145	
	v, *t*_0_	−101	
	a, v, *t*_0_	−229	
	a	v	*t*_0_
SS	2.57(0.29)	5.05(0.39)	0.18(0.01)
RS	3.06(0.32)	5.52(0.40)	0.15(0.02)
Group Variation	1.36(0.21)	1.58(0.20)	0.06(0.01)
p(high > low)	0.90	0.81	0.92

Bottom Parameter estimates from (a, v, t) model, group variability, and the probability that the greater value between SS and RS is indeed greater for each of the parameters (i.e. *p*(*a*_*RS*_ > *a*_*SS*_); *p*(*v*_*RS*_ > *v*_*SS*_); 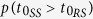.
